# Performance of pre-hospital evaluations in ruling out invasive chest stab wounds

**DOI:** 10.1186/s13049-020-00725-w

**Published:** 2020-05-05

**Authors:** Pascal Augustin, Elise Guivarch, Quentin Pellenc, Yves Castier, Sandrine Boudinet, Sebastien Tanaka, Philippe Montravers, Alexy Tran-Dinh

**Affiliations:** 1 Department of Anesthesia and Intensive Care, Groupe Hospitalier Bichat Claude Bernard, Assistance Publique Hôpitaux de Paris, 46 rue Henri Huchard, 75018 Paris, France; 2 Department of Thoracic and Vascular Surgery, Groupe Hospitalier Bichat Claude Bernard, Assistance Publique Hôpitaux de Paris, Paris, France; 3 Université Paris VII Diderot, Sorbonne Paris Cité, Paris, France; 4 INSERM U1188, La Réunion, France; 5 INSERM U1152, Paris, France; 6 INSERM U1148, Paris, France

**Keywords:** Chest penetrating trauma, Stab, Overtriage

## Abstract

**Background:**

Some guidelines advocate for managing patients with penetrating thoracic wounds in trauma centres with cardiothoracic surgery. This systematic approach is questionable. Only 15% of these patients require surgery. It is known that clinical examination fails to detect hemopneumothorax in penetrating trauma. However, no studies have evaluated the combined diagnostic performance of vital signs and the clinical evaluation of wounds. The clinical characteristics of wounds have not been investigated. We aimed to evaluate the ability of combinations of pre-hospital signs to rule out invasive chest stab trauma.

**Methods:**

This was a prospective observational study. All consecutive adult patients hospitalized in the perioperative acute care unit of a tertiary university hospital were included. Injury diagnoses were provided by exploratory surgery and imaging tests. Patients with a final diagnosis of invasive wounds (IWs) and patients with only superficial wounds were compared. Data regarding management and outcome were analysed.

**Results:**

A total of 153 patients were included. After imaging or surgery, 58 (38%) patients were diagnosed with only superficial wounds, and 95 (62%) were diagnosed with thoracic or abdominal IWs. The false-negative rate of pre-hospital evaluations in the diagnosis of IWs was 42% [31–51]_IQR25–75_. In stable patients, pre-hospital data could not rule out IWs, with a negative predictive value of 58% and a positive predictive value of 70%. Twenty-nine (19%) patients required early emergent cardiothoracic surgery. Among these patients, 8 (28%) had no evidence of IWs in the pre-hospital period. Among the 59 patients without pre-hospital signs of IWs, 19 (33%) underwent at least one emergent procedure.

**Conclusions:**

The combination of pre-hospital vital signs, visual evaluation of wounds, and physical examination failed to rule out IWs in patients with chest stab wounds. This implies that caution is needed in triage decision-making.

## Background

The Centers for Disease Control and Prevention guidelines advocate for the management of patients with penetrating chest trauma in high-level trauma institutions with cardiothoracic surgery facilities [1]. In contrast, the French guidelines do not provide such formal recommendations regarding this issue [2]. They advocate for transfer to specialized trauma centres for unstable patients. For stable patients, there is no clear position statement [2]. The term “penetrating” includes all stab wounds caused by low-velocity sharp weapons [3]. In cases of chest stab trauma, the “penetrating trauma” may not have truly penetrated beyond the chest muscle wall and thus may not have caused organ injuries. In the context of stab and gunshot chest wounds, the older literature differentiated between effectively penetrating trauma and superficial trauma [4, 5]. In more recent literature dealing with penetrating chest injuries, there is no classification differentiating actual invasive chest wounds and superficial wounds [6, 7]. Therefore, even stable patients with seemingly superficial chest stab trauma are labelled as having “penetrating chest trauma”.

The earliest data show that physical examination may not reliably detect the presence of haemothorax or pneumothorax in cases of stab trauma [8–10]. However, some studies suggest that clinical exploration of the wound provides valuable information about the wound depth [11, 12]. No study has evaluated the capability of a pre-hospital evaluation involving vital signs, wound characteristics, and physician judgement to reliably distinguish patients with invasive stab wounds from patients with superficial stab wounds. Invasive wounds (IWs) can be suspected based on an inspection of the wound or general signs, such as a drop in haemoglobin levels or the presence of haemodynamic and respiratory dysfunction.

Admitting all patients with chest stab trauma in high-level trauma centres may result in overtriage. In fact, some patients are discharged from the hospital a few hours after their admission [5, 7]. Only 5 to 15% of patients require emergent cardiothoracic surgery [6, 13], and overtriage is associated with work overload [14]. However, delaying transfer to a specialized centre and the performing of surgery by a non-cardiothoracic surgeon may affect the survival of patients, even in cases of initially occult injuries [15–17].

The main objective of the present study was to assess the effectiveness of a pre-hospital evaluation to assess and rule out IWs in patients with chest stab trauma. The secondary objectives were to evaluate pre-hospital factors that could discriminate patients with IWs from others, to describe injury diagnoses, and, finally, to assess invasive procedures that were performed in patients with and without pre-hospital signs suggestive of IWs.

## Methods

### Study design

We conducted a monocentre prospective observational study in the perioperative acute care unit (PACU) of a tertiary university hospital in Paris, France. Patients were prospectively included from January 2013 to December 2015. The study protocol was approved by the ethics committee of Ile de France 1 (IRB no. 00008522). Patients were informed of the nature of study. All consecutive patients referred to the PACU were included. The exclusion criteria were patients under the age of 18 years and secondary transfers from other hospitals.

### Pre-hospital management

The study was observational, and there was no modification of patient management. The pre-hospital medical assessment was performed by the physician from the mobile medical ICU ambulance (MICA), SAMU in French. The MICA team was led by a senior physician specialized in emergency medicine. Measurement of venous haemoglobin was systematically performed using a haemoglobinometer. No other blood sample analysis was performed systematically. The MICA team stabilized patients and decided which institution each patient needed to be referred to. They may also have performed a needle decompression or chest tube insertion if needed.

### Management at admission

For the first assessment, all patients underwent a chest X-ray and blood tests upon admission to the PACU, except when they needed immediate surgery. Transthoracic echocardiography (TTE) and thoracoabdominal computed tomography (CT) scans were performed based on the decision of the medical team. If pneumothorax was diagnosed at the first assessment, a chest tube was inserted prior to other imaging tests or prior to transfer to the operating room. In the case of haemothorax, a CT scan was systematically performed. In stable patients with a normal chest X-ray, a CT scan could be performed to rule out occult injuries [7]. Unstable or agonic patients were directly transferred to the operating room for emergent sternotomy or thoracotomy.

Protocol for CT scans:

Thorax and abdomen were assessed by 64-row multidetector CT with a slice thickness of 0.625 mm. The CT protocol included unenhanced, arterial, and portal vein phases. A senior radiologist, an intensivist, and a surgeon interpreted the CT scans.

### Data collection

On admission, information provided by the MICA team was recorded. The main reason for transfer to the PACU of our specialized centre was recorded and identified as follows:

- On a systematic basis (seemingly superficial wound and normal vital signs)
- Suspected IW by MICA team physician
- Signs of circulatory dysfunction
- Signs of ventilatory dysfunction
- Drop in haematocrit level

Other pre-hospital data were recorded at the first assessment by the MICA team and during transportation. Upon hospital admission, the following data were recorded: vital signs and biological data, Injury Severity Score (ISS) [18], Abbreviated Injury Scale maximal (AIS max) values [19], and visual characteristics of the wounds (number, size, location, blowing/subcutaneous emphysema, haemorrhagic wound, and associated extrathoracic wounds).

**Fig. 1:**
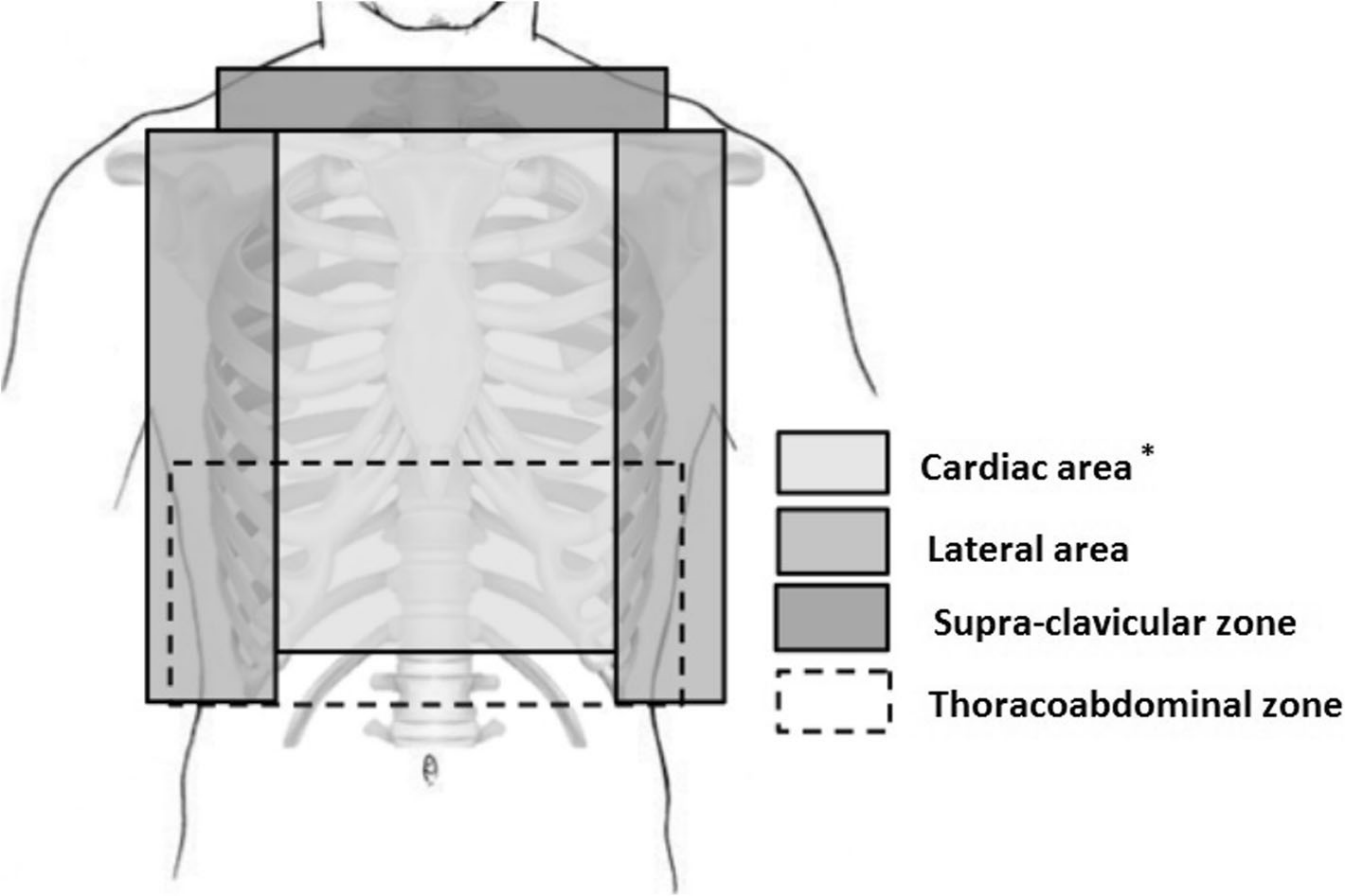
Representation of the thoracic regions

Figure 1 shows how wounds were classified according to their location. A wound could be classified in more than one category. This classification was chosen in accordance with previous studies [3, 7, 20, 21]. Data from imaging and/or surgery allowed for the classification of wounds as superficial or invasive, as defined in Table1. Classifications and definitions are given in Table 1. We recorded the definitive diagnosis, management (need for chest drainage, cardiothoracic surgery, and abdominal explorations), and outcome data.

**Table 1:** Definitions from the methods section

Thoracic invasive wound	Injuries beyond the chest wall muscles (diaphragm, thoracic organs, or ribs)
Abdominal invasive wound	Intra-abdominal organs or diaphragm injuries
All-type invasive wound	Thoracic or abdominal injuries
Superficial wound	No injury beyond wall muscles
No pre-hospital sign of an invasive wound	Normal vital signs, no drop in haemoglobin level, AND apparent superficial wound at physical examination performed by the MICA physician
Pre-hospital signs of invasive wounds	Apparent invasive wound, drop in haemoglobin level, OR cardio-respiratory dysfunction
Correctly classified wound	Pre-hospital suspicion of invasive wound concordant with the definitive diagnosis
Stable patients	No organ dysfunction or drop in haemoglobin level
Cardiovascular dysfunction	Pulse > 110 bpm or mean blood pressure < 60 mmHg
Respiratory dysfunction	Respiratory rate > 25/min or pulse oximetry < 95%
Life-saving surgery	A surgery performed for a potentially fatal organ injury
Overtriage [14]	Rate of patients without any intervention

Definition: *MICA* Mobile ICU Ambulance

### Outcome

The primary outcome was the false-negative rate of the pre-hospital evaluation for the diagnosis of IWs. The negative predictive value, positive predictive value, and Youden index were calculated to assess the performance of the pre-hospital evaluation in the diagnosis of IWs in all patients and stable patients specifically. Secondary outcomes were the rate of need for emergency surgery or chest drainage in all patients, and in patients without pre-hospital signs of IW.

### Statistical analysis

Categorical variables were expressed as numbers and percentages. Continuous variables were expressed as the means and 95% confidence intervals (95% CIs) for normally distributed variables and medians and interquartile ranges (IQRs) [25–75] for other variables. The normal distribution of continuous variables was assessed by histograms and QQ plots. We calculated positive and negative predictive values, which are reported as percentages and 95% CIs. Categorical variables were compared using a chi-squared test or Fisher’s exact test, and continuous variables were compared using the Mann-Whitney U test or t-test, as appropriate. Statistical analyses were performed with R software 3.4.2 (The R Foundation for Statistical Computing, Vienna, Austria).

## Results

### General characteristics

During the study period, 153 patients were included. There were 139 male patients (93% of total), with a median age of 29 years old [22–39]IQR25–75, and they were admitted during the night shift in 59% of cases. The main reasons for transfer to the PACU are given in Table 2. New features observed upon hospital admission are given in Table 2. After imaging tests or surgery, 58 patients (38%) were diagnosed with only superficial thoracic wounds, and 95 (62%) had thoracic or abdominal IWs. Sixty-four patients had extrathoracic wounds, including 23 (15%), 24 (16%), and 45 (29%) patients with abdominal, cervicofacial, and extremity wounds, respectively. With respect to extrathoracic wounds, the median of the highest AIS was 1 [1–2]IQR25–75, and the median ISS wounds was 2 [1–5]IQR25–75.

**Table 2:** Pre-hospital assessment and final diagnosis of IWs for stable and unstable patients

	Stable patients (*n* = 119)		Unstable patients (*n* = 34)
	Suspicion of supetavrficial wounds (*n* = 59)	Suspicion of invasive wounds (*n* = 60)	
Superficial wound (*n* = 58)^a^	34	18	6
Invasive wound (*n* = 95)^a^	25	42	28

Definition: *IW* Invasive Wound

^a^Definitive diagnosis

### Main results

Table 3 provides data regarding the suspected and actual IWs for stable and unstable patients. In the 119 stable patients, we found 48 superficial wounds (44%). The false-negative rate of the pre-hospital evaluation for the diagnosis of IWs was 42% [30 –- 54]_95% CI._ In the whole population, the negative predictive value was 58% [46 – 70]_95% CI_, the positive predictive value was 74% [65 – 83]_95% CI_, and the Youden index was 0.32. In the 119 stable patients, the negative predictive value was 58% [46 – 70]_95% CI_, the positive predictive value was 70% [58 – 82] _95% CI_, and the Youden index was 0.28.

**Table 3:** Characteristics of patients with and without a final diagnosis of IWs

	IW	Superficial W	p**
	*(n* = 95)	(*n* = 58)	
No pre-hospital sign of IW, n (%)	25(26)	34(59)	< 0.01^a^
Correctly classified, n (%)	70(74)	34(59)	0.05^a^
AIS max, med [IQR,25–75%]	3[3–4]	1[1–3]	< 0.01^b^
ISS, med [IQR,25–75%]	9[9–16]	2[1–4]	< 0.01^b^
Pre-hospital Pulse: first (bpm), mean[95% CI]	97[92–102]	99[94–104]	0.52^c^
Pre-hospital Pulse: maximum (bpm), mean[95% CI]	101[97–106]	103[99–107]	0.23^c^
Pulse at hospital admission (bpm), mean[95% CI]	104[100–107]	97[93–101]	< 0.01^c^
Pre-hospital SBP: first (mmHg), mean[95% CI]	115[110–120]	125[119–130]	0.02^c^
Pre-hospital SBP: minimum (mmHg), med [IQR]	110[90–121]	121[100–131]	< 0.01^b^
SBP at hospital admission (mmHg), med [IQR]	126[110–140]	125[117–142]	0.40^b^
Pre-hospital RR first (/min), med [IQR]	20|18–25]	20[16–25]	0.74^b^
Pre-hospital RR: minimum (/min), med [IQR]	20[16–21]	20[18–25]	0.64^b^
RR at hospital admission (/min), med [IQR]	20[17–25]	18[15–21]	0.04^b^
Pre-hospital Hb level: first (g/L), mean[95% CI]	139[136–143]	144[140–148]	0.13^c^
Pre-hospital Hb level: Min (g/L), mean[95% CI]	135[131–139]	139[134–144]	0.1^c^
Hb level on admission (g/L), med [IQR]	131[122–146]	134[117–143]	0.26^b^
Pulse oximetry (%): first, med [IQR]	99[97–100]	99[98–100]	0.38^b^
Pulse oximetry at hospital admission (%), med [IQR]	99[98–100]	100[97–100]	0.07^b^
Location of wound			
Cardiac box, n (%)	60(63)	41(71)	0.34^a^
Lateral zone, n (%)	25(26)	41(71)	0.995^a^
Thoracoabdominal zone, n (%)	60(63)	32(55)	0.328^a^
Posterior zone, n (%)	19(20)	18(31)	0.122^a^
Supraclavicular zone, n	4	1	
Number of wounds, med [IQR]	1 [1–2]	1[1–2]	0.5^b^
Extrathoracic wounds, n (%)	35(37)	29(50)	0.11^a^
Abdominal wounds^a^, n (%)	13(17)	10(14)	0.55^a^
Cervicofacial wounds, n (%)	14(18)	10(14)	0.68^a^
Extremity wounds, n (%)	24(25)	21(36)	0.15^a^

Definition: ^a^ thoracoabdominal wounds excluded; *AIS* Abbreviated Injury Scale, *ISS* Injury Severity Score, *SBP* Systolic Blood Pressure, *RR* Respiratory Rate, *Hb* Haemoglobin

**: *p*-value for comparison between IWs and superficial wounds

Statistical tests: b = chi-squared test;c = Mann-Witney U test; c = t test

### Factors associated with the presence of invasive wounds

Table 4 compares pre-hospital data for patients with a final diagnosis of all-type IWs (thoracic or abdominal) to those with only superficial wounds. Patients with IWs more frequently had signs of cardiovascular or respiratory dysfunction than other patients. There was no significant difference regarding the location of wounds. There were 77 patients (50%) with thoracic IWs. When comparing patients with and without thoracic IWs, we found similar results (data not shown). All 255 wounds were compiled. Individual anatomical characteristics were evaluated for all-type IWs and non-IWs are shown in the supplemental data (Table S1). We found 36 abdominal IWs, 89 thoracic IWs, and 12 combined thoracic/abdominal IWs. The characteristics of thoracic IWs are given in the supplemental data (Table S2).

**Table 4:** Anatomical characteristic of all-types non-invasive and invasive wounds

	All-types IW (*n*=113)	Non IW (***n***=141)	*p*
Number of wounds, med[IQR]	1[1-2]	3[1-4]	<0.01^a^
Blowing wound/SE, n (%)	16(14)	1(0.7)	<0.01^b^
Hemorrhagic wound, n (%)	14(12)	10(7)	0.16^b^
Wound size (centimeters), med[IQR]	2[1.5-3]	1.5[1-2.6]	<0.01^a^
Location of the wounds			
Thoracoabdominal zone, n (%)	66(58)	63(45)	0.02^b^
Cardiac box, n (%)	67(59)	95(67)	0.34
Supraclavicular zone, n (%)	3(3)	4(3)	1^c^
Lateral zone, n (%)	42(37)	47(33)	0.44^b^
Posterior wound, n (%)	21(19)	57(40)	<0.01

Definition : *IW* Invasive Wound, *SE* Subcutaneous Emphysema * The same woundassociated with thoracic and abdominal lesions Statistical tests: ^a^ = Mann-Withney U-test; ^b^ = Chi-square test; ^c^ = Fisher's exact test

### Management and outcome

During the pre-hospital period, cardiopulmonary resuscitation was performed in six patients, who subsequently arrived at the PACU with no pulse. One patient was declared dead upon admission. Eight patients had been intubated for either organ failure or agitation/analgesia. One patient had received a pre-hospital needle decompression, and one had undergone chest tube drainage. Seven patients did not have blood samples because of immediate emergent surgery. Table 5 summarizes the investigations performed on admission for patients with and without pre-hospital signs of IWs.

**Table 5:** Anatomical characteristic of non-invasive wounds and invasive thoracic wounds.

	Thoracic IW (*n*=89)	No thoracic IW (*n*=166)	p
Blowing wound/SE, n (%)	16(18)	1(0.6)	<0.01^a^
Hemorrhagic wound, n (%)	14(16)	10(6)	1^b^
Wound size, med[IQR]	2[1-3]	1[1.5-3]	0.81^c^
Wound location			
Cardiac box, n (%)	50(56)	112(67)	0.07^b^
Thoracoabdominal zone, n (%)	43(52)	86(52)	0.59^b^
Supraclavicular zone, n (%)	3(3)	4(2.5)	0.65^a^
Lateral zone, n (%)	36(40)	53(32)	0.17^b^
Posterior wound, n (%)	19(21)	59(36)	0.03^b^

Definition: IW= Invasive Wound; SE: Subcutaneous Emphysema; IQR=Interquartiel Range 25-75%. Statistical tests: ^a^ = Fisher's exact test; ^b^ = Chi squar test; ^c^ = Mann-Withney U-test.

Table 6 gives data on the management of patients with and without pre-hospital signs of IWs. Seventy-four (48%) patients had no intervention (overtriage). Fifty-eight (38%) patients underwent cardiothoracic or abdominal surgery, of which 16 (28%) showed no pre-hospital clinical signs of IWs (Table 7). Twenty-nine (19%) patients required cardiothoracic surgery. Of these patients, eight (28%) showed no pre-hospital evidence of IWs. Among the 59 patients without pre-hospital signs of IWs, 19 (33%) underwent at least one procedure, including 16 all-type surgical procedures (eight cardiothoracic surgeries and eight abdominal surgeries) and six chest tube insertions. Forty-eight patients (31%) underwent surgery within two hours of admission, including 23 patients (15%) who underwent cardiothoracic surgery. Of the 59 patients with no pre-hospital signs of IWs, 13 patients underwent a procedure for all-type IWs within two hours, including chest tube insertion in five patients and cardiothoracic surgery in four patients.

**Table 6:** Imaging tests performed at admission

	Pre-hospital signs of IWs (*n* = 94)	No signs of IWs (*n* = 59)	p*
No investigation, n	3	0	
Chest X-ray, n (%)	83 (88)	59(100)	< 0.01^a^
Chest X-ray alone, n (%)	8(9)	9(15)	0.19^a^
TTE, n (%)	64(68)	35(59)	0.27^a^
TTE alone, n (%)	7(7)	0	0.03^b^
CT scan, n (%)	53(56)	45(76)	0.013^a^
CT scan alone, n	0	0	
Chest X-ray and TTE, n (%)	23(24)	5(8)	0.01^a^
TTE and CT scan, n	1	0	
Chest X-ray and CT scan, n (%)	19(20)	15(25)	0.45^a^
Chest X-ray TTE, CT scan, n (%)	33(35)	30(51)	0.06^a^

Definition: *IW* invasive wound, *TTE* transthoracic echocardiography, *CT* computed tomography

*: p-value for comparison between patients with pre-hospital signs of IWs and others

Statistical tests: ^a^ = chi-squared test; ^b^ = Fisher’s exact test

**Table 7:** Management and outcomes for patients with and without signs of invasive wounds

	Pre-hospital signs of IWs (*n* = 94)	No signs of IWs (*n* = 59)	p**
No intervention, n (%)	34(64)	40(32)	< 0.01^a^
Cardiothoracic surgery or chest tube, n (%)	42(45)	12(20)	< 0.01^a^
Chest tube, n (%)	28(30)	6(10)	< 0.01^a^
All-type surgery, n (%)	42(45)	16(27)	0.03^a^
Cardiothoracic surgery, n (%)	21(22)	8(14)	0.18^a^
Sternotomy, n (%)	7(7)	3(5)	0.57^b^
Thoracotomy, n (%)	17(18)	5(8)	0.10^a^
Cardiothoracic surgery alone, n (%)	16(17)	8(14)	0.57^a^
Abdominal procedure^a^, n (%)	24(26)	8(14)	0.13^a^
Abdominal procedure alone^a^, n (%)	17(18)	8(14)	0.46^a^
Abdominal and cardiothoracic surgery, n (%)	3(3)	0	0.07^b^
Life-saving surgery, n (%)	30(32)	10(17)	0.04^a^
Surgery within 2 h of admission, n (%)	38(40)	10(17)	0.02^a^
Intubation for all causes, n (%)	48(51)	23(39)	0.20^a^
Intubation after arrival at hospital, n (%)	40(43	23(39)	0.66^a^
Time to surgery** (from admission), med [IQR]	1.5[1–2]	2[1.9–4.3]	0.01^c^
Lactate blood level: peak (mmol/L), med [IQR]	2.3[1–3]	2[1.5–3]	0.53^c^
LOS in PACU, med [IQR]	2[1–2]	1[1–2]	0.50^c^
Discharge from PACU to home, n (%)	20(23)	23(40)	0.04^a^
Hospital LOS, med [IQR]	5[3–9]	3[2–6]	< 0.01^c^
Transfusion, n (%)	17(18)	2(3)	0.02^a^
Haemoglobin level: minimum (g/L), med [IQR]	120[105–130]	128[115–138]	0.02^c^
Death, n (%)	6(6)	0	0.12^b^

Definitions: *LOS* Length of Stay, *PACU* Perioperative Acute Care Unit, *IQR* Interquartile Range 25–75%

^a^: Laparoscopy or laparotomy, including exploratory surgery

**: p-value for comparison between patients with signs of IWs and others

Statistical tests: ^b^ = chi-squared test; ^c^ = Fisher’s exact test; ^d^ = Mann-Witney U test

Data on outcomes are displayed in Table 4. The in-hospital mortality rate was 4%. Forty percent of patients without pre-hospital signs of IWs were discharged directly from the PACU to home.

## Discussion

The present study found a 42% rate of false-negative pre-hospital evaluations for the diagnosis of IWs after chest stab trauma. The negative predictive value was 58%. The pre-hospital clinical examination associated with physician judgement failed to reliably rule out IWs after chest stab trauma. Therefore, it does not appear to be possible to predict the need for emergent cardiothoracic procedures at the pre-hospital stage. This finding is in accordance with those of the few previous studies that have addressed this issue [8–10]. The prospective study by Bokhari, which was conducted in more than 600 patients, including 153 penetrating chest traumas, showed that in penetrating trauma, the accuracy of physical examination in detecting haemopneumothorax was low. In the study, which focused on stable patients, only three diagnostic criteria were evaluated independently [8]. The retrospective study by Kong including 405 stable patients with penetrating chest wounds, also showed a poor performance of the physical examination for the diagnosis of a significant injury. The negative predictive value was 47% [9]. Although these studies did not have a strictly identical endpoint, their results were similar and they also concluded that physical examination cannot rule out an invasive wounds [8, 9]. Our study not only analysed stable patients but also aimed to integrate physical examinations, visual judgement, and vital signs. This is the first study showing that even when combining vital signs, physical examinations, and visual characteristics of wounds, the pre-hospital evaluation missed many IWs. The high rate of false-negative pre-hospital evaluations encourages us to conclude that all chest stab wounds should be considered invasive until the results of imaging tests are obtained.

The CDC guidelines advocate for the transfer of all patients with penetrating chest trauma in trauma institutions with cardiothoracic surgery facilities [1]. The French guidelines do not provide such formal recommendations [2]. These two guidelines are mainly based on expert opinions [1, 2]. Only the Western Trauma Association guidelines provide a single reference backing their statement position [6, 22], indicating a lack of data relevant to this issue. Our study brings useful data.

In our study, while patients with IWs had lower blood pressure than other patients, at the pre-hospital stage other pre-hospital vital signs, or the location of wounds, were not discriminating. Even though this result regarding pre-hospital blood pressure is not surprising, we expected pulse rate to be an early sign of IWs. The pain or anxiety associated with the context of chest stab wounds, even in patients with no serious injury, could be an explanation for this conflicting result [23]. Due to this lack of discriminating criteria in the univariable analysis, we did not perform a multivariable analysis by logistic regression.

We found a 58% rate of superficial wounds in patients without pre-hospital signs of IWs and a rate of 44% in all stable patients. This result shows a relatively high rate of overtriage in stable patients. Only one study has attempted to assess diagnosis in stable patients specifically [5]. In this retrospective Spanish study comprising 90 penetrating chest injuries, including 77 cases of stabbing, there were 29 (38%) superficial wounds [5]. In our study, we found that 40% of patients were discharged directly to their homes, which is comparable to the 38% of patients without surgery or chest drainage in the Spanish study [5].

In the present study, 15% of all patients needed cardiothoracic surgery within two hours of admission. Furthermore, 17% of patients with no pre-hospital signs of IWs underwent a surgical procedure within two hours, and 17% underwent life-saving surgery. These results are in accordance with previous studies showing that the need for emergent thoracotomy was approximately 10 to 20% within the first 24 h [6, 13]. The study by Onat et al. is the only recent paper that provides rates of surgery in chest penetrating trauma [13]. The study comprised more than 1000 patients with gunshot or stab wounds, with a 15% rate of surgery [13]. Their study design does not allow us to obtain the specific rate of thoracotomy in the subgroup of patients with stabs or the specific rate of surgery in initially stable patients [13]. Despite this data showing rates of surgery to be approximately 15%, which may be low, it is difficult to risk undertriage in absence of reliable criteria for ruling out IWs.

Although the data are scarce, previous studies and the present study are fairly consistent in their encouraging of overtriage. The overtriage target of the American College of Surgeons Committee on Trauma is below 35% [14]. Given our definition, overtriage is estimated to be 48% in the present study. Although overtriage may induce logistical problems, including work overload in the trauma units of specialized centres or high costs, some data show that outcome is improved when emergent thoracotomy is performed by a cardiothoracic surgeon [15]. A medico-economic study would be of interest to help balance the cost of overtriage with the drawbacks of undertriage.

Some limitations exist in the present study. First, the signs of cardiovascular and respiratory dysfunction may only be related to agitation or pain in the context of stab trauma. Second, the decisions of the pre-hospital medical team may evolve between the first and final triage decisions because of alterations in patients’ condition. It is difficult to reliably know the main reason for triage decisions; decisions in triage are often multifactorial. Finally, this is a monocentre study that is very dependent on the local logistical organization. However, we have shown that our results are in accordance with those of previous studies, suggesting that our population may not be peculiar. Further studies may evaluate the impact of the extended focused assessment with sonography for trauma (e-FAST) in decision-making [24].

## Conclusions

The combination of physical examination, vital signs, and visual characteristics of the wounds cannot reliably rule out IWs in patients with chest stab trauma. Seventeen percent of patients without signs of IWs needed cardiothoracic surgery within two hours of admission. Based on this study, even stable patients should be referred to acute care facilities with cardiac or thoracic surgery. Nevertheless, these results should be confirmed in future studies.

## Supplementary information

**Additional file 1 Table S1**. Anatomical characteristics of all types of non-invasive and invasive wounds.

**Additional file 2 Table S2**. Anatomical characteristic of non-invasive wounds and invasive thoracic wounds.

## Data Availability

The datasets analysed during the current study are available from the corresponding author on request.

## Abbreviations

IW: Invasive wound
PACU: Perioperative acute care unit
MICA: Mobile medical ICU ambulance
TTE: Transthoracic echocardiography
CT: Computed tomography
ISS: Injury Severity Score
AIS: Abbreviated Injury Scale
E-FAST: Extended-Focused Assessment with Sonography in Trauma
IQR: Interquartile range
CI: Confidence Interval
