# Supplementary material for: Performance of pre-hospital evaluations in ruling out invasive chest stab wounds
Source: Scand J Trauma Resusc Emerg Med. 2020 May 5;28:33. doi: 10.1186/s13049-020-00725-w (PMC7201546; doi:10.1186/s13049-020-00725-w)
Supplement: Supplementary file 1 — Additional file 1 Table S1. Anatomical characteristics of all types of non-invasive and invasive wounds. [file 13049_2020_725_MOESM1_ESM.doc]

| Table S1. Anatomical characteristics of all types of non-invasive and invasive wounds. | | | | |
| --- | --- | --- | --- | --- |
|  | All-types IW  (n=113) | Non-IW  (n=141) | p* | |
| Number of wounds, med[IQR] | 1[1-2] | 3[1-4] | <0.01a | |
| Blowing wound/SE, n (%) | 16(14) | 1(0.7) | <0.01b | |
| Haemorrhagic wound, n (%) | 14(12) | 10(7) | 0.16b | |
| Wound size (centimetres), med[IQR] | 2[1.5-3] | 1.5[1-2.6] | <0.01a | |
| Location of the wounds |  |  |  | |
| Thoracoabdominal zone, n (%) | 66(58) | 63(45) | 0.02b | |
| Cardiac box, n (%) | 67(59) | 95(67) | 0.34 | |
| Supraclavicular zone, n (%) | 3(3) | 4(3) | 1c | |
| Lateral zone, n (%) | 42(37) | 47(33) | 0.44b | |
| Posterior wound, n (%) | 21(19) | 57(40) | <0.01b | |
| Definition: IW= Invasive Wound; SE=Subcutaneous Emphysema  The same wound associated with thoracic and abdominal injuries  *: p-value for comparison between IWs and non-IWs  Statistical tests: a = Mann-Witney U test; b = chi-squared test; c = Fisher's exact test | | | |  |
