# Supplementary material for: Performance of pre-hospital evaluations in ruling out invasive chest stab wounds
Source: Scand J Trauma Resusc Emerg Med. 2020 May 5;28:33. doi: 10.1186/s13049-020-00725-w (PMC7201546; doi:10.1186/s13049-020-00725-w)
Supplement: Supplementary file 2 — Additional file 2 Table S2. Anatomical characteristic of non-invasive wounds and invasive thoracic wounds. [file 13049_2020_725_MOESM2_ESM.doc]

| Table S2. Anatomical characteristic of non-invasive wounds and invasive thoracic wounds. | | | |
| --- | --- | --- | --- |
|  | Thoracic IW (n=89) | Thoracic non-IW (n=166) | p* |
| Blowing wound/SE, n (%) | 16(18) | 1(0.6) | <0.01a |
| Haemorrhagic wound, n (%) | 14(16) | 10(6) | 1b |
| Wound size, med[IQR] | 2[1-3] | 1[1.5-3] | 0.81c |
| Wound location |  |  |  |
| Cardiac box, n (%) | 50(56) | 112(67) | 0.07b |
| Thoracoabdominal zone, n (%) | 43(52) | 86(52) | 0.59b |
| Supraclavicular zone, n (%) | 3(3) | 4(2.5) | 0.65a |
| Lateral zone, n (%) | 36(40) | 53(32) | 0.17b |
| Posterior wound, n (%) | 19(21) | 59(36) | 0.03b |
| Definition: IW= Invasive Wound; SE= Subcutaneous Emphysema; IQR= Interquartile Range 25-75  *: p-value for comparison between thoracic IW and No thoracic IW  Statistical tests: a = Fisher's exact test; b = chi-squared test; c = Mann-Witney U-test. | | | |
